# Supplementary material for: The SMIM25-COX-2 Axis Modulates the Immunosuppressive Tumor Microenvironment and Predicts Immunotherapy Response in Hepatocellular Carcinoma
Source: Curr Issues Mol Biol. 2025 Aug 27;47(9):693. doi: 10.3390/cimb47090693 (PMC12468620; doi:10.3390/cimb47090693)
Supplement: Supplementary file 1 [file cimb-47-00693-s001.zip › Supplementary Figures.pdf]

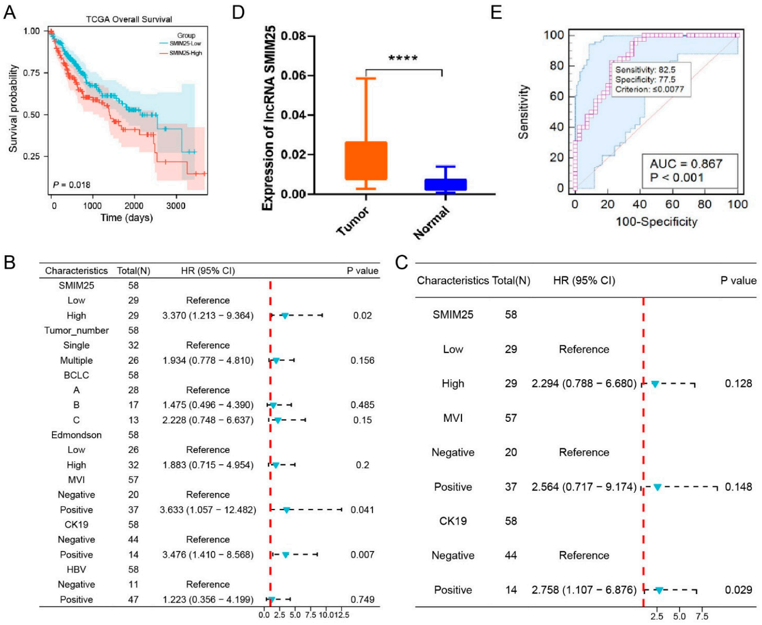

Figure S1. Clinical significance of expressions of SMIM25 in peripheral blood in HCC. (**A**) Overall survival curves illustrate the overall survival of for patients in the TCGA cohort stratified by the expression of SMIM25. (**B**) Univariate Cox regression analysis (overall survival) in the Guangxi HCC cohort 1. (**C**) Multivariate Cox regression analysis (overall survival) in the Guangxi HCC cohort 1. (**D**) Boxplot illustrates the expression of SMIM25 in the whole blood of HCC patients and healthy volunteers. (**E**) The corresponding ROC curve based on SMIM25 expression. **** $p < 0.0001$.

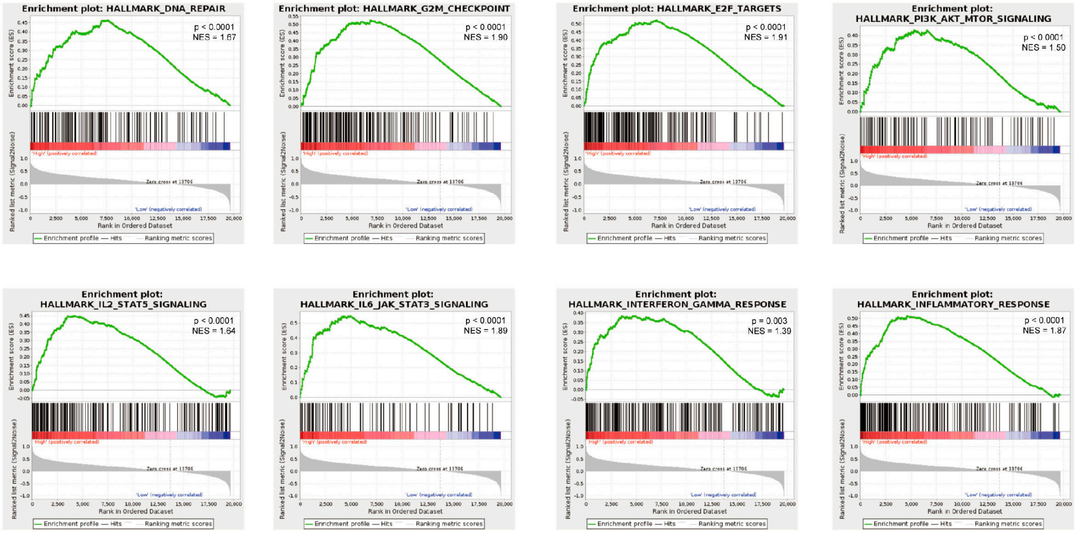

Figure S2. Gene set enrichment analysis of DEGs in HCC patients between high and low SMIM25 expression. Gene Set Enrichment Analysis (GSEA) plots show the enrichment of hallmark gene sets in HCC with high expression of SMIM25.

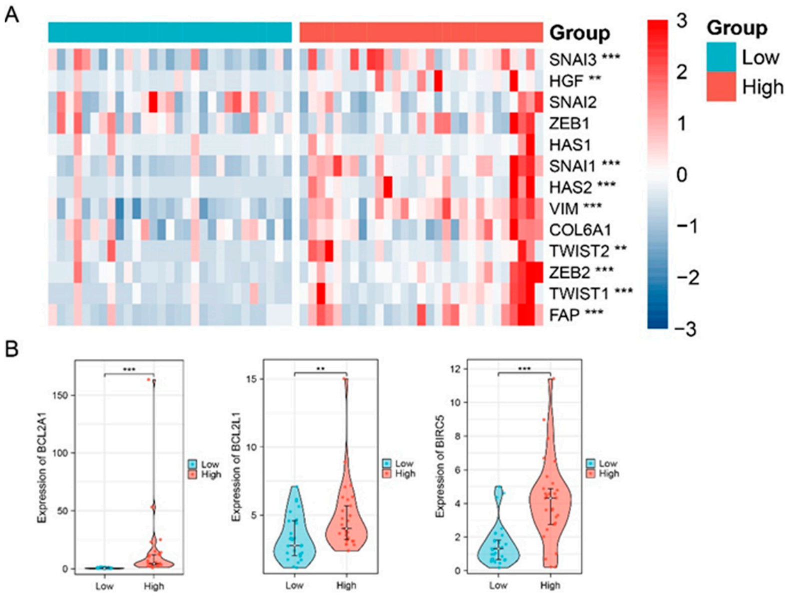

Figure S3. Differential Expression Analysis of EMT-Related and Apoptosis Genes in SMIM25-High versus SMIM25-Low Groups. (**A**) The heatmap illustrates the expression levels of EMT-related genes in the SMIM25-high and SMIM25-low groups. (**B**) Violin plots display the expression levels of apoptosis-related genes in the SMIM25-high and SMIM25-low groups. ** $p < 0.01$; *** $p < 0.001$.

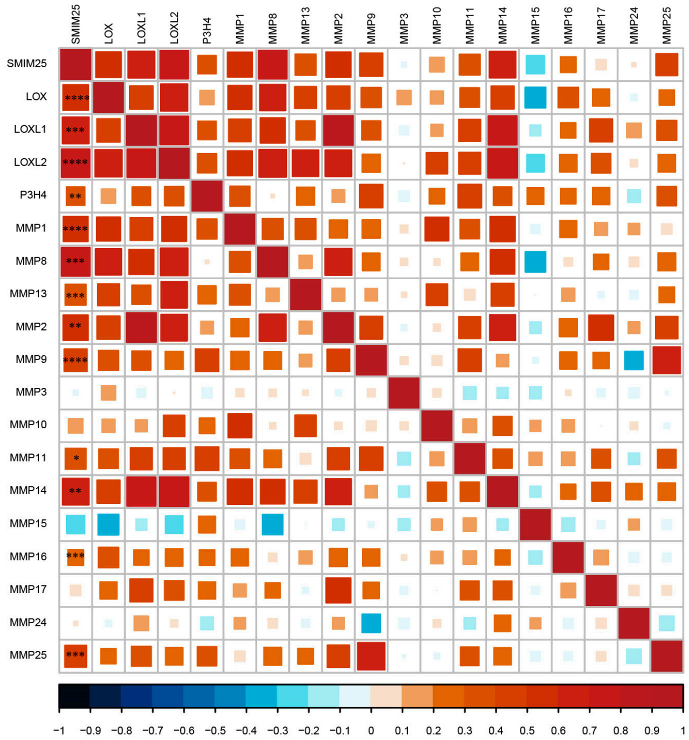

Figure S4. Correlation analysis between SMIM25 and collagen-modifying genes. The correlation heatmap shows the correlation between the expression of SMIM25 and the expression of collagen-modifying genes. * $p < 0.05$; ** $p < 0.01$; *** $p < 0.001$; **** $p < 0.0001$.

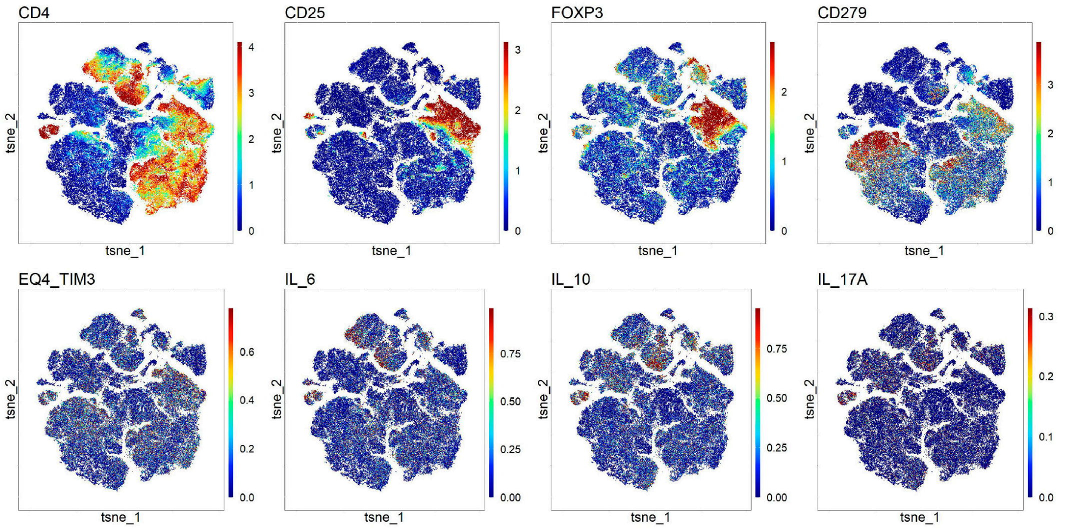

Figure S5. Expression analysis of immune-related markers in Cluster 13. t-SNE plot visualizing the expression patterns of immune markers CD4, CD25, Foxp3, CD279, TIM_3, and IL_6, IL_10, IL_17A. Color gradient indicates normalized expression levels.

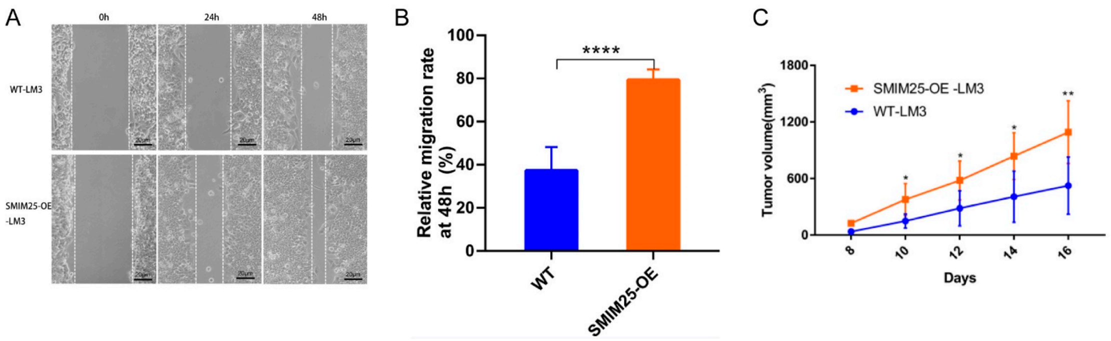

Figure S6. Overexpression of SMIM25 alters biological capabilities in HCC Cell Line. (**A**,**B**) The migration ability of both wild-type LM3 cells (WT-LM3) and LM3 cells with SMIM25 overexpression (SMIM25-OE-LM3) is assessed through Scratch migration assay. (**C**) Tumor volume is compared in nude mice implanted with either WT-LM3 or SMIM25-OE-LM3 cells. * $p < 0.05$; ** $p < 0.01$; **** $p < 0.0001$.

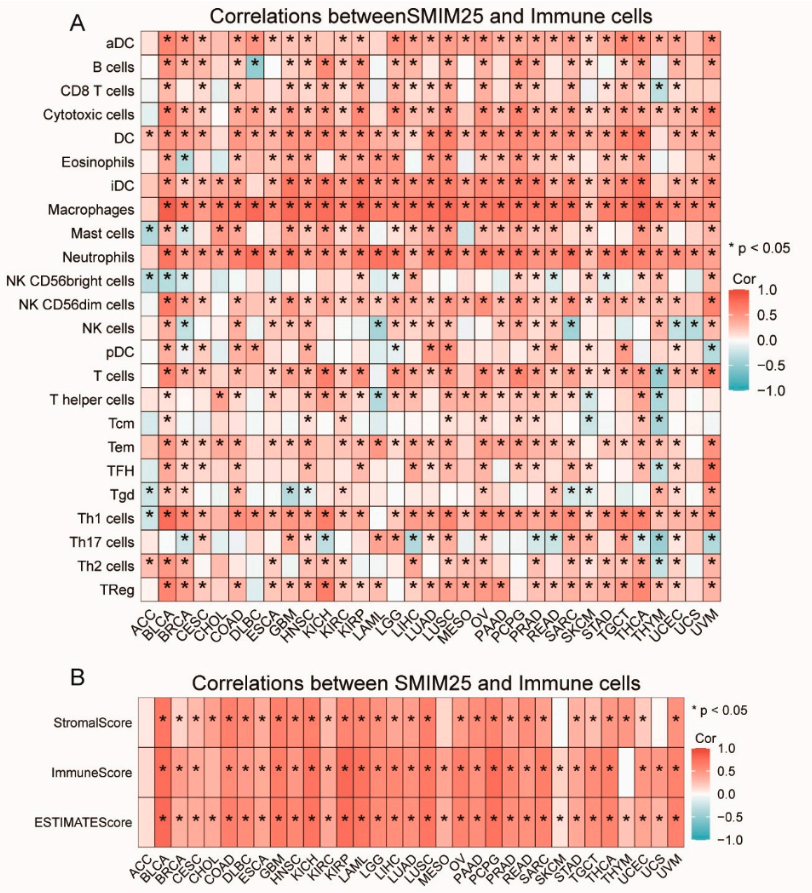

Figure S7. Correlation analysis between SMIM25 expression and immune infiltration across most cancer types. (**A**,**B**) ssGSEA (**A**) and ESTIMATE (**B**) algorithms are used to calculate the correlation between SMIM25 expression and immune infiltration in all tumor types from TCGA. * $p < 0.05$.
