# Supplementary material for: The SMIM25-COX-2 Axis Modulates the Immunosuppressive Tumor Microenvironment and Predicts Immunotherapy Response in Hepatocellular Carcinoma
Source: Curr Issues Mol Biol. 2025 Aug 27;47(9):693. doi: 10.3390/cimb47090693 (PMC12468620; doi:10.3390/cimb47090693)
Supplement: Supplementary file 1 [file cimb-47-00693-s001.zip › Supplementary Table S1.pdf]

Supplementary Table S1. CyTOF Antibody Panel

Source	Identifier	Antibodies	Clone	Metal
Fluidigm	3142001B	CD19	HIB19	142Nd
Fluidigm	3145010B	CD163	GHI/61	145Nd
Fluidigm	3148010B	CD14	RMO52	148Nd
Fluidigm	3146014B	CD11c	3.9	146Nd
Fluidigm	3176022A	CD196/CCR6	G034E3	176Yb
Fluidigm	3164009B	CD161	HP-3G10	164Dy
Fluidigm	3150017B	CD27	LG.3A10	150Nd
Fluidigm	3168008B	CD206/MMR	15-2	168Er
Fluidigm	3149010B	CD25/IL-2R	2A3	149Sm
Fluidigm	3154003B	CD3	UCHT1	154Sm
Fluidigm	3141006B	CD326/EpCAM	9C4	141Pr
Fluidigm	3174004B	CD4	SK3	174Yb
Fluidigm	3089003B	CD45	HI30	89Y
Fluidigm	3170010B	CD45RA	HI100	170Er
Fluidigm	3162023B	CD66b	80H3	162Dy
Fluidigm	3173005B	HLA-DR	L243	173Yb
Fluidigm	3175017B	CD274/PD-L1	29E.2A3	175Lu
Fluidigm	3156008B	CD86	IT2.2	156Gd
Fluidigm	3165037B	CD223/LAG-3	11C3C65	165Ho
Fluidigm	3153008B	TIM-3	F38-2E2	153Eu
Fluidigm	3159028A	Foxp3	259D/C7	159Tb
Fluidigm	3167009A	CD197/CCR7	G043H7	167Er
Fluidigm	3171002B	Granzyme B	GB11	171Yb
Fluidigm	3155009B	CD279/PD-1	EH12.2H7	155Gd
Fluidigm	3147002B	IL-6	MQ2-13A5	147Sm
Fluidigm	3158017B	IFN-r	B27	158Gd
Fluidigm	3169006B	IL-17A	BL168	169Tm
Fluidigm	3172024B	Ki-67	B56	172Yb
Fluidigm	3166008B	IL-10	JES3-9D7	166Er
Fluidigm	3163010B	TGFbeta	TW4-6H10	163Dy
Fluidigm	3152002B	TNFa	Mab11	152Sm
Biolegend	302302	CD20	2H7	161Dy
Biolegend	303502	CD38	HIT2	143Nd
Biolegend	304202	CD45RO	UCHL1	151Eu
Biolegend	301002	CD8a	RPA-T8	144Nd
