# Supplementary material for: The SMIM25-COX-2 Axis Modulates the Immunosuppressive Tumor Microenvironment and Predicts Immunotherapy Response in Hepatocellular Carcinoma
Source: Curr Issues Mol Biol. 2025 Aug 27;47(9):693. doi: 10.3390/cimb47090693 (PMC12468620; doi:10.3390/cimb47090693)
Supplement: Supplementary file 1 [file cimb-47-00693-s001.zip › Supplementary Table S2.pdf]

Supplementary Table S2 IMC Antibody Panel

Antibodies	Label /Metal	Clone	Source/ Company	Product_ID /Identifier
Foxp3	155Gd	Rabbit	NOUVS	NB100-39002
CD44	153Eu	Rabbit	Abcam	ab157107
CD326/EpCAM	150Nd	Rabbit	Abcam	ab71916
HepPar-1	176Yb	OCH1E5	Abcam	ab234028
CD133	172Yb	Rabbit	Abcam	ab16518
CD31	156Gd	JC/70A	Abcam	ab9498
CD90	163Dy	7E1B11	Abcam	ab181469
YAP1	149Sm	Mouse	Abcam	ab56701
PD_L1	145Nd	73-10	Abcam	ab226766
CD68	141Pr	KPI	Abcam	ab213096
Pan_Cytokeratin	160Gd	c-11	Abcam	ab7753
Cytokeratin_7	151Eu	RCK105	Abcam	ab9021
CK19	173Yb	A53-B/A2	BioLegend	628502
CD4	159Tb	RPA-T4	BioLegend	300502
a_SMA	142Nd	Polyclonal	Abcam	ab5694
CD45	152Sm	CD45-2B11	Fluidigm	3152016D
Beta_Catenin	165Ho	D13A1	Fluidigm	3165032D
E_Cadherin	158Gd	24E10	Fluidigm	3158029D
Vimentin	143Nd	RV202	Fluidigm	3143029D
CD20	161Dy	H1	Fluidigm	3161029D
CD8a	162Dy	C8/144B	Fluidigm	3162034D
GranzymeB	167Er	EPR20129-217	Fluidigm	3167021D
Collagen_I	169Tm	Polyclonal	Fluidigm	3169023D
CD3	170Er	Polyclonal	Fluidigm	3170019D
CD66a	171Yb	CD66a-B1.1	Fluidigm	3171020D
Ki_67	175Lu	Rabbit	Abcam	ab15580
CD34	154Sm	Rabbit	Abcam	ab8158
FAP	147Sm	Rabbit	Abcam	ab53066
Arginase	164Dy	Rabbit	NOUVS	NBP1_32731
